# Glucokinase Regulatory Protein (GCKR) Links Metabolic Reprogramming With Immune Exclusion: Insights From a Pan-Cancer Analysis and Gastric Cancer Validation

**DOI:** 10.1155/humu/4240223

**Published:** 2025-11-05

**Authors:** Shaohua Fan, Youfu He, Zhen Chen, Chiting Yuan, Jiangjie Chen, Chenhao Xu, Weixing Huang, Can Yao, Dun Hong, Liwei Zhang

**Affiliations:** ^1^Department of Orthopedics, Taizhou Hospital of Zhejiang Province, Zhejiang University School of Medicine, Taizhou, China; ^2^Department of Cardiology, Guizhou Provincial People's Hospital, Guiyang, Guizhou Province, China; ^3^Institute of Bone Metabolism, Taizhou Hospital of Zhejiang Province, Zhejiang University School of Medicine, Taizhou, China; ^4^Department of General Surgery, Taizhou Hospital of Zhejiang Province, Zhejiang University School of Medicine, Taizhou, China

**Keywords:** CAF, gastric cancer, GCKR, immune microenvironment, metabolic reprogramming, multiomics, prognosis, spatial transcriptomics

## Abstract

Glucokinase regulatory protein (GCKR) is a metabolic regulator implicated in glucose homeostasis, but its genetic and functional roles in cancer remain poorly understood. Through integrated pan-cancer multiomics and experimental analyses, we mapped the expression and mutational landscape of GCKR with a focus on gastric cancer. GCKR expression was downregulated in most tumors but upregulated in subsets such as kidney renal papillary carcinoma (KIRP) and lung adenocarcinoma (LUAD). Genomic profiling revealed recurrent alterations, with the highest mutation frequencies observed in sarcoma (SARC) and uterine corpus endometrial carcinoma (UCEC), and missense mutations representing the predominant variant type, particularly in breast cancer (BRCA). Functionally, reduced GCKR expression in gastric cancer was associated with an immune-cold phenotype characterized by diminished cytotoxic T cell infiltration, impaired antigen presentation, and metabolic reprogramming. Spatial transcriptomics and single-cell analyses highlighted compartment-specific heterogeneity and links with cancer-associated fibroblasts and macrophages. Clinically, low GCKR expression predicted poorer survival and reduced immunotherapy benefit, while higher expression indicated selective sensitivity to MEK inhibitors including refametinib and PD0325901. These findings define GCKR as both a mutation- and expression-driven biomarker that connects metabolic regulation with immune remodeling, offering translational value for prognosis and precision therapy in gastric cancer.

## 1. Introduction

Cancer arises from somatic cells that progressively accumulate genetic mutations, and its underlying mechanisms are highly complex [1]. It continues to affect millions of people annually, and despite advances in chemotherapy, cancer-related mortality remains unacceptably high [2]. Tumor immunotherapy, especially the use of immune checkpoint inhibitors (ICIs), has achieved encouraging clinical outcomes in selected patients [3]. However, durable responses to immunotherapy are observed only in a subset of patients, as most solid tumors display an “immune-cold” phenotype [4], characterized by a lack of immune cell infiltration in the tumor microenvironment, with T cell dysfunction or suppression [5]. This attenuated immune response state is a major barrier to the success of current tumor immunotherapy. Therefore, understanding the key factors that regulate the tumor immune microenvironment is crucial for identifying new therapeutic targets and enhancing the response rates to immunotherapy.

The importance of metabolism in the carcinogenesis process is well established [6]. Cancer cells undergo significant metabolic reprogramming to support their rapid growth and survival [7]. The discovery of the Warburg effect has further highlighted that cancer cells preferentially utilize glycolysis to generate energy. As a result, research into cancer glycolysis has become an important part of cancer studies [8]. In parallel, advances in whole-genome sequencing have significantly improved our understanding of tumor molecular mechanisms, underscoring the value of pan-cancer analyses that link gene expression with clinical prognosis and underlying biological pathways [9].

Glucokinase regulatory protein (GCKR) is a metabolic regulator predominantly expressed in the liver and is traditionally thought to be involved in maintaining glucose metabolic homeostasis [10]. Recent research suggests that metabolic reprogramming is closely linked to tumor immunity. Disruptions in glucose metabolism not only promote tumor cell growth but may also regulate the functional state of immune cells in the tumor microenvironment by affecting their metabolic activity [11, 12]. As a key metabolic node, GCKR's potential role in tumors has garnered increasing attention. Numerous studies have also confirmed that GCKR is closely related to the development of diabetes and nonalcoholic fatty liver disease [13, 14]. Nevertheless, whether GCKR contributes to regulating the tumor immune microenvironment or influences responses to immunotherapy remains unresolved.

Using a pan-cancer analysis strategy [15], this study systematically characterizes the expression pattern of GCKR across multiple tumor types, evaluates its prognostic significance, and explores its association with the immune microenvironment. We also validate its potential clinical significance in predicting immunotherapy responses using multiple independent immunotherapy cohorts. Additionally, we investigate potential interventions targeting GCKR through drug-sensitivity analysis and small-molecule drug screening. Overall, this research aims to elucidate the functional mechanisms of GCKR in the “immune-cold” state and provide a theoretical basis for the development of novel tumor immunotherapy targets.

## 2. Methods

### 2.1. Data Collection and Processing

The Cancer Genome Atlas (TCGA, http://cancergenome.nih.gov/) and Genotype-Tissue Expression (GTEx, https://gtexportal.org/home/) databases were used to obtain pan-cancer data on GCKR expression [16]. GCKR expression profiles in normal human tissues and cancer cell lines were derived from the Human Protein Atlas (HPA, https://www.proteinatlas.org/) [17]. The mutation frequency of GCKR in TCGA cohorts was calculated using the cBioPortal database (https://www.cbioportal.org/) [18]. Pan-cancer analysis tools were from the online bioinformatics tool SangerBox3.0 (http://sangerbox.com/). The TIMER2.0 database (http://timer.cistrome.org) with seven immune infiltration algorithms was used to calculate the association between GCKR and the tumor immune microenvironment [19]. Chemotherapy-related data were obtained from the GDSC database (https://www.cancerrxgene.org) [20].

### 2.2. Expression and Variation Analysis

The “Wilcox” signed-rank test was used to calculate expression differences between tumor and normal tissues. The “gganatogram” R package was used to visualize GCKR expression in different organs of the human body. GCKR mutation data were obtained from the cBioPortal website (http://www.cbioportal.org) [18].

### 2.3. Diagnosis and Prognosis

The potential role and significance of GCKR in pan-cancer diagnosis were evaluated using the pROC package. The “survminer” R package was used to describe the relationship between GCKR expression and prognostic indicators, including overall survival (OS), disease-specific survival (DSS), progression-free interval (PFI), and disease-free interval (DFI). Through the Kaplan–Meier (KM) analysis, we assessed whether GCKR was a protective or risk factor, and the prognostic value was validated in external datasets from the GEO database using univariate Cox proportional hazards regression models [21].

### 2.4. Pathway and Mechanism Analysis

The “GSVA” R package was used for *z*-score standardized analysis of 14 functional genomes, and Pearson's correlation coefficients were used to assess the relationship between each genome and GCKR expression. Tumor samples were divided into high expression (top 30%) and low expression (bottom 30%) groups based on GCKR expression levels. Gene Set Enrichment Analysis (GSEA) was used to analyze the differential regulatory patterns of pan-cancer and characteristic genes, as well as metabolism-related gene sets.

### 2.5. Tumor Immune Infiltration and Immune Cells

Spearman's rank correlation analysis was used to analyze stem cell properties of pan-cancer samples. Seven algorithms (CIBERSORT, CIBERSORT-ABS, EPIC, MCP-COUNTER, quanTIseq, TIMER, and xCELL) were used for immune cell infiltration scoring and tumor purity assessment. We systematically verified the regulatory relationship between GCKR expression and immune-related gene sets (chemokines, chemokine receptors, immune stimulatory molecules, immunosuppressive factors, and MHC molecules) and analyzed its role in the tumor immune microenvironment.

We used immunological infiltration data assessed by multiple algorithms through TIMER2.0 (https://timer.cistrome.org/) [19], combined with transcriptome data, to examine the association between GCKR expression in specific cell types and survival or clinical outcomes in patients. Patients were divided into four groups based on the mean expression of GCKR and the mean level of cancer-associated fibroblast (CAF) infiltration [22]. KM survival analysis was conducted using the survfit function from the survival package [23], and the log-rank test was applied to assess both pairwise and overall significance among the four groups.

### 2.6. Analysis of Gastric Cancer Single-Cell Dataset

We used data provided by GSE167297 in the TISCH2 database using the Sparkle database (https://grswsci.top) for relevant analyses [24]. The TISCH2 database (http://tisch.comp-genomics.org/) collects single-cell RNA-seq (scRNA-seq) data resources from human and mouse tumors. Using the Kruskal–Wallis rank-sum test (Kruskal's test), we evaluated the expression differences of GCKR in different cell types. Based on whether GCKR was expressed, all cells were divided into expression-positive and expression-negative groups, and the limma package was used to compare score differences between the two groups. The AUCell package was used to evaluate relevant biological pathways [25].

### 2.7. Spatial Transcriptome Analysis of the Relationship Between GCKR and Immune Cells

Spatial transcriptome analysis was performed using the Sparkle database (https://grswsci.top/) [24] and SpatialTME (https://www.spatialtme.yelab.site/). The most abundant cell type in each region was calculated, and the SpatialDimPlot function in the Seurat package was used to visualize the maximum cell composition of each region [26]. The SpatialFeaturePlot function in the Seurat package was used to visualize GCKR expression in each region. Meanwhile, correlation analysis was used to calculate the correlation between cell content and cell content in all SPOTs, as well as the correlation between cell content and GCKR expression, with visualization completed using the LINKET package.

### 2.8. Machine Learning

To further evaluate the predictive significance of GCKR, we applied 10 machine learning algorithms, including random forest (RF), support vector machine (SVM), gradient boosting machine (GBM), k-nearest neighbor (KNN), logistic regression (Logit), generalized linear model (GLM), stepwise linear discriminant analysis (stepLDA), partial least squares (PLS), Naive Bayes, and Elastic Net.

Model performance was assessed using receiver operating characteristic (ROC) curves and the area under the curve (AUC). To examine the robustness of predictions, residual distributions were analyzed by reverse cumulative distribution plots and boxplots. Feature importance was further calculated across models to evaluate the contribution of GCKR compared with other cancer-related genes.

All analyses were performed in R (Version 4.3.2). The main packages included glmnet for Lasso and Elastic Net, randomForest for RF, e1071 for SVM and Naive Bayes, gbm for GBM, class for KNN, MASS for stepLDA, and pls for PLS. ROC curves and AUC values were computed using the pROC package, and residual analyses were visualized using base R and ggplot2.

### 2.9. Immunohistochemistry (IHC) Analysis

This study used IHC to detect GCKR expression in gastric cancer tissues and adjacent tissues. The total of nine gastric cancer tissue samples were acquired from the Taizhou Hospital of Zhejiang Province (*n* = 6) and Guizhou Provincial People's Hospital (*n* = 3). All participants signed and completed a form indicating their informed consent, and the ethical approval agency was the Taizhou Hospital of Zhejiang Province and Guizhou Provincial People's Hospital. Tissue sections were stained using conventional immunohistochemical staining methods. ImageJ (V1.8) software was used for data statistical analysis, and GraphPad Prism 10.4.2 software was used for graphing and statistical analysis to evaluate the intensity and distribution of GCKR expression.

### 2.10. Enzyme-Linked Immunosorbent Assay (ELISA) Analysis

We used the ELISA kit from Assay Genie (Cat. No.: HUEB1443) to quantitatively measure GCKR protein expression levels in gastric cancer tissues and adjacent normal tissues. Tissue samples were homogenized under ice-bath conditions using a tissue homogenizer, and total protein was extracted using RIPA lysis buffer (containing protease and phosphatase inhibitors). Protein concentrations were determined using a BCA protein quantification kit, and all samples were adjusted to the same concentration. We strictly followed the manufacturer's protocol for the Assay Genie ELISA kit (HUEB1443) and measured the absorbance of each well at 450 nm using a microplate reader. GCKR protein concentrations in each sample were calculated based on the standard curve and expressed as picograms per milligram total protein. Each sample was tested in triplicate, and the average values were used for statistical analysis. Paired *t*-tests were used to compare GCKR protein expression levels between gastric cancer tissues and adjacent normal tissues, with *p* < 0.05 considered statistically significant.

### 2.11. Quantitative Real-Time PCR (qRT-PCR)

Total RNA was extracted using TRIzol reagent (Invitrogen), then reverse-transcribed into complementary DNA (cDNA) with the PrimeScript RT Kit (Takara). Quantitative PCR amplification was performed using SYBR Green Master Mix (Vazyme). The relative expression levels of target mRNAs were determined using the 2^−ΔΔCt^ method, with *β*-actin serving as the internal control. The primer sequences were as follows:

*β*-Actin-forward: 5⁣′-CTCTTCCAGCCTTCCTTCCT-3⁣′.

*β*-Actin-reverse: 5⁣′-AGCACTGTGTTGGCGTACAG-3⁣′.

GCKR-F: 5⁣′-AGTGAACAGCTGCCTTTGTG-3⁣′

GCKR-R: 5⁣′-TCCAGGTCTGTGTAGGATGG-3⁣′

### 2.12. Western Blotting

Proteins were extracted from the treated cells and quantified. Equal amounts of total protein were loaded and separated via SDS-PAGE using the Mini-PROTEAN Tetra Cell System (Bio-Rad). The proteins were then transferred to a polyvinylidene difluoride (PVDF) membrane. After transfer, the membrane was blocked with Protein-Free Rapid Blocking Buffer (PS108P, Epizyme, China) for 30 min at room temperature. Subsequently, the membrane was incubated overnight at 4°C with the appropriate primary antibody (ab154120, Abcam, United States). The next day, it was washed and incubated with the corresponding secondary antibody for 1 h at room temperature. Protein bands were visualized using BeyoECL Plus chemiluminescence reagent (P0018S, Beyotime, China).

### 2.13. Statistical Analysis

All statistical analyses were performed using GraphPad Prism 10.4.2 (GraphPad Software, LLC, San Diego, CA, United States). Each experiment was independently repeated at least three times, and data are presented as the mean ± standard deviation (SD). Differences between two groups were assessed using a two-tailed Student's *t*-test. Bioinformatics analysis and data visualization were conducted in R (Version 4.3.2). A *p* value less than 0.05 was considered statistically significant.

## 3. Results

### 3.1. Expression and Mutation of GCKR in Cancer

**Figure 1:**
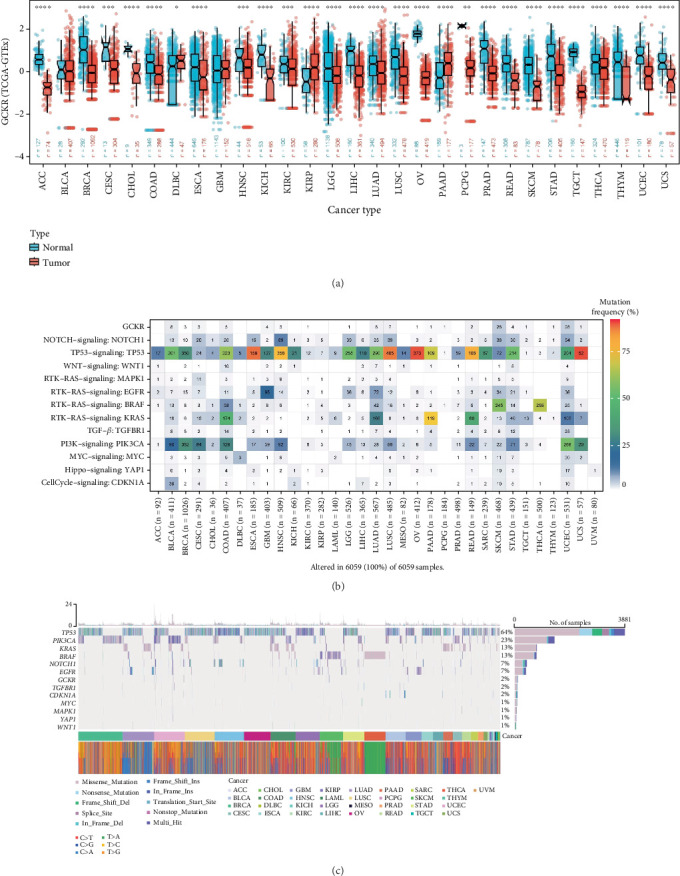
GCKR expression and mutation in cancer. (a) GCKR expression in tumor versus normal tissues (TCGA+GTEx). (b) Distribution of GCKR and other cancer-related signals. (c) Mutation sites and frequency of GCKR in cancer. Note: ⁣^∗∗∗∗^*p* < 0.0001, ⁣^∗∗∗^*p* < 0.001, ⁣^∗∗^*p* < 0.01, and ⁣^∗^*p* < 0.05.

To investigate the differential expression of GCKR between tumor and normal tissues, we analyzed unpaired samples from the TCGA and TCGA–GTEx databases. The results revealed significant differences in GCKR expression between tumor and normal tissues across various cancers. In most cases, GCKR expression was significantly higher in normal tissues compared to tumor tissues (e.g., breast cancer [BRCA], cholangiocarcinoma [CHOL], HNSC, and KICH), while only a few cancers such as kidney renal papillary cell carcinoma (KIRP), lung adenocarcinoma (LUAD), and THCA showed elevated GCKR expression in tumor tissues (Figure 1a and Supporting Information 1: Figure S1A). We further visualized GCKR expression across human organs (Supporting Information 1: Figure S1B), showing higher expression in normal tissues of organs like the breast, liver, and pancreas. In contrast, its expression was generally lower in tumor tissues, except in the pancreas and gallbladder where slight elevation was observed. Next, we examined GCKR mutation profiles. Mutation rates of GCKR and other common oncogenes/tumor suppressors across various cancers indicated relatively high mutation frequencies in sarcoma (SARC) and uterine corpus endometrial carcinoma (UCEC) (Figure 1b). Analysis of mutation types showed that missense mutations were the most frequent single nucleotide variant (SNV) type in both GCKR and other cancer-related genes, with BRCA exhibiting the highest mutation rate (Figure 1c).

### 3.2. Machine Learning Validation of the Significance of GCKR in Gastric Cancer

**Figure 2:**
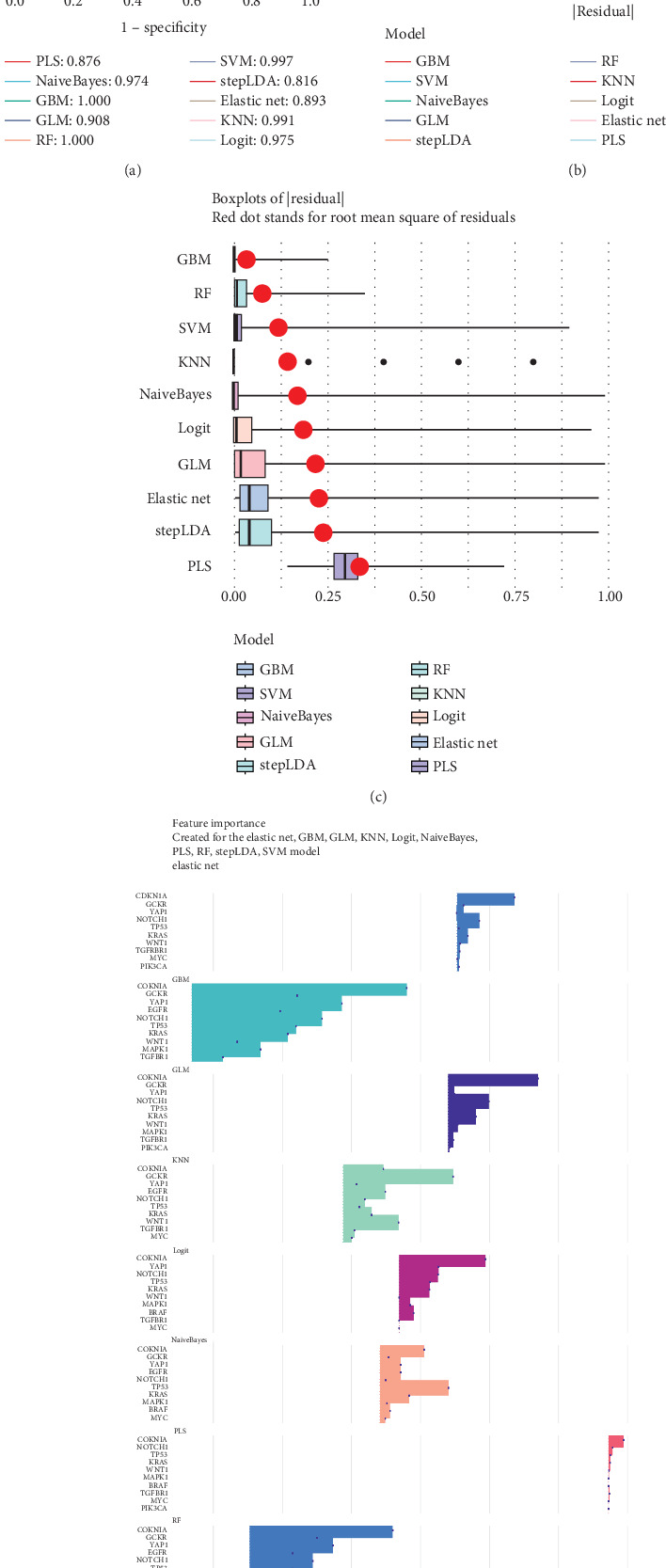
Machine learning validation of GCKR in gastric cancer. (a) ROC curves of 10 machine learning models. (b) Reverse cumulative distribution of residuals. (c) Boxplots of residuals. (d) Feature importance ranking across models.

We applied 10 machine learning algorithms to validate the predictive value of GCKR. The integrated ROC analysis showed that most models exhibited high discriminative performance, with AUC values ranging from 0.816 to 1.000 (Figure 2a). Among them, RF and GBM achieved perfect classification, while SVM and KNN also performed with near-perfect accuracy. Residual distribution analysis confirmed the robustness of these models. Both the cumulative distribution curves and the boxplots demonstrated that RF, GBM, and SVM maintained the lowest residuals, whereas stepLDA and PLS showed relatively weaker performance (Figure 2b,c). Feature importance analysis further revealed that GCKR consistently ranked among the top predictors together with classical oncogenes such as TP53, KRAS, and CDKN1A (Figure 2d). Additional machine learning validation, including Boruta, Lasso, SVM-RFE, and XGBoost, is provided in Supporting Information 2: Figures S2A, S2B, S2C, S2D, and S2E.

### 3.3. Experimental Validation of GCKR Expression in Gastric Cancer

**Figure 3:**
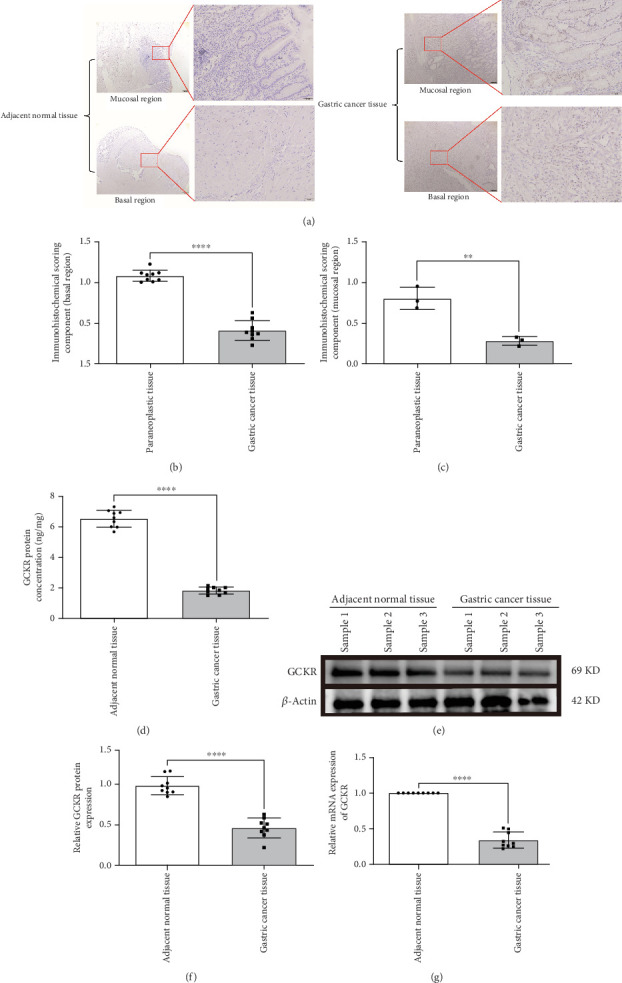
GCKR expression in gastric cancer. (a–c) IHC validation of GCKR in tumor versus adjacent tissue. (d) ELISA analysis. (e, f) Western blot results. (g) RT-qPCR data. Note: ⁣^∗∗∗∗^*p* < 0.0001 and ⁣^∗∗∗^*p* < 0.01.

IHC confirmed lower GCKR expression in gastric cancer tissues compared to adjacent nontumor tissues (Figures 3a, 3b, and 3c), particularly in mucosal and basal regions (*p* < 0.05). ELISA results also indicated significantly lower GCKR levels in gastric cancer (Figure 3d, *p* < 0.05). Western blotting further validated the downregulation of GCKR protein in tumor tissues (Figure 3e,f), and qRT-PCR confirmed reduced mRNA expression levels in gastric cancer samples (Figure 3g).

### 3.4. Association Between GCKR Expression and Clinical Features

**Figure 4:**
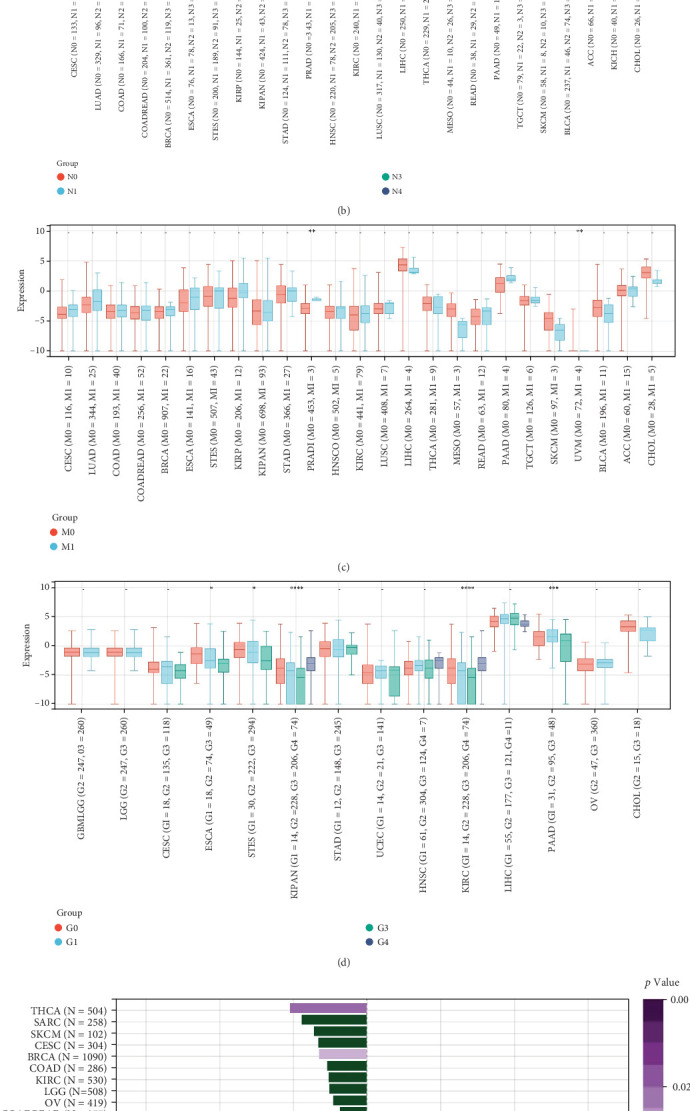
Correlations between GCKR expression and clinical features. (a) GCKR and tumor T stage. (b) GCKR and N stage. (c) GCKR and M stage. (d) GCKR and overall tumor stage. (e) GCKR and patient age. Note: ⁣^∗∗∗∗^*p* < 0.0001, ⁣^∗∗∗^*p* < 0.001, ⁣^∗∗^*p* < 0.01, and ⁣^∗^*p* < 0.05.

We next explored the relationship between GCKR expression and clinical parameters such as tumor stage, patient age, and sex. GCKR expression was significantly associated with T stage in ESCA and KIPAN (Figure 4a) and with N stage in KIPAN and THCA, showing increased expression with advancing stage (Figure 4b). In PRAD and UVM, GCKR was correlated with M stage (Figure 4c). Furthermore, in KIPAN, KIRC, and PAAD, GCKR expression was positively associated with pathological grade, indicating a potential role in tumor progression (Supporting Information 3: Figure S3A). A similar trend was observed with overall stage in ESCA, KIPAN, and PAAD (Figure 4d). No significant correlation was found between GCKR expression and sex (Supporting Information 3: Figure S3B). However, we observed an inverse correlation between GCKR and age in THCA and BRCA and a positive correlation in ESCA, PCPG, and STES (Figure 4e).

### 3.5. Clinical Diagnostic and Prognostic Value of GCKR

**Figure 5:**
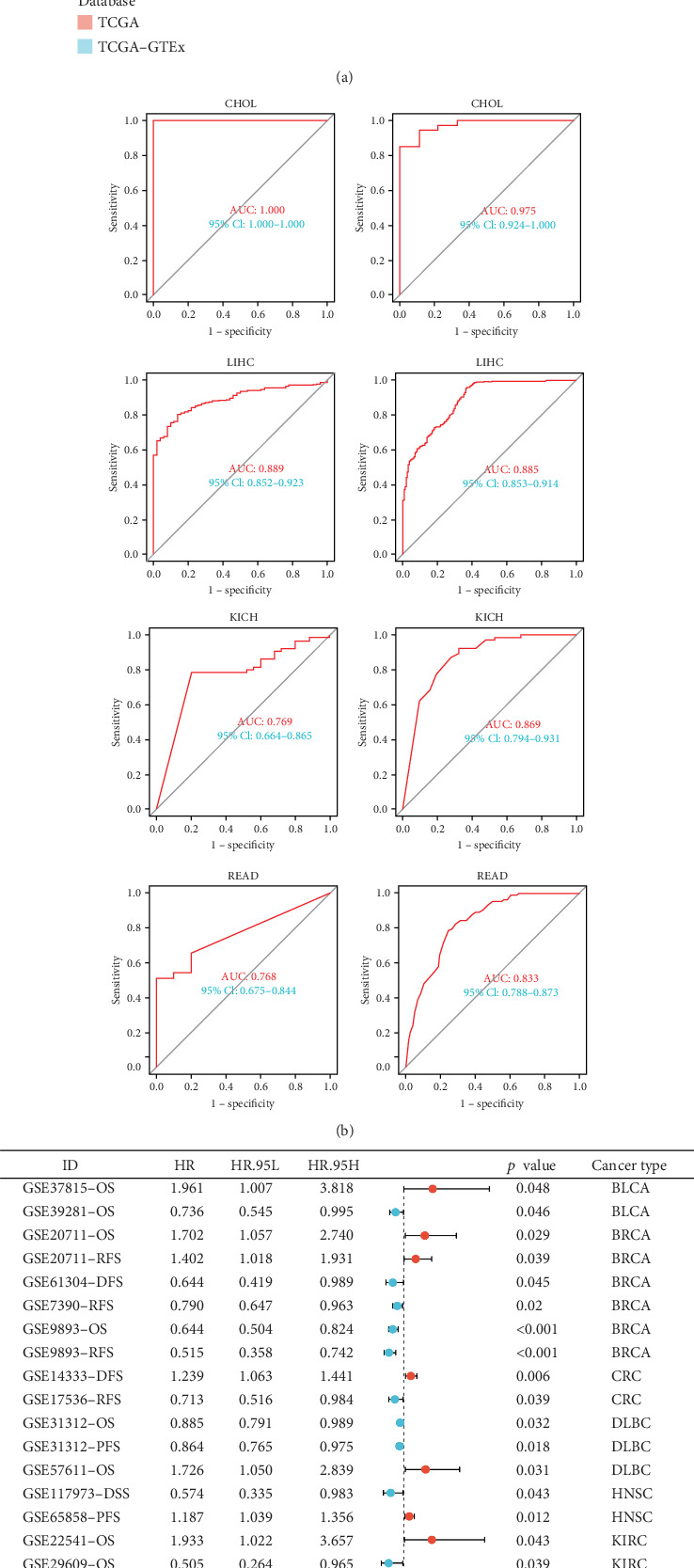
Clinical diagnostic and prognostic value of GCKR across cancers and independent cohorts. (a) AUC values from TCGA and TCGA+GTEx. (b) ROC curves in CHOL, LIHC, and READ. (c) Univariate survival analysis showing correlation with prognosis.

To explore the clinical relevance of GCKR, we first evaluated its diagnostic performance using TCGA and TCGA–GTEx datasets. ROC curve analyses revealed that GCKR effectively distinguished tumor from normal tissues in several cancers, including CHOL, liver hepatocellular carcinoma (LIHC), and rectum adenocarcinoma (READ), with AUC values approaching or exceeding 0.85 (Figure 5a,b). In gastric cancer, consistent downregulation of GCKR expression further supported its diagnostic utility.

We then assessed the prognostic implications of GCKR in independent GEO and ICGC cohorts. Meta-analysis demonstrated that GCKR expression was significantly associated with patient outcomes, including OS, disease-free survival (DFS), relapse-free survival (RFS), and progression-free survival (PFS). Notably, in BRCA, diffuse large B-cell lymphoma (DLBC), and LUAD, high GCKR expression predicted unfavorable outcomes, whereas in gastric cancer, reduced GCKR expression was correlated with poorer prognosis (Figure 5c). These findings underscore the dual clinical value of GCKR, highlighting its potential both as a diagnostic marker and as a prognostic biomarker for patient stratification.

### 3.6. Association of GCKR With Cancer-Related Pathways

**Figure 6:**
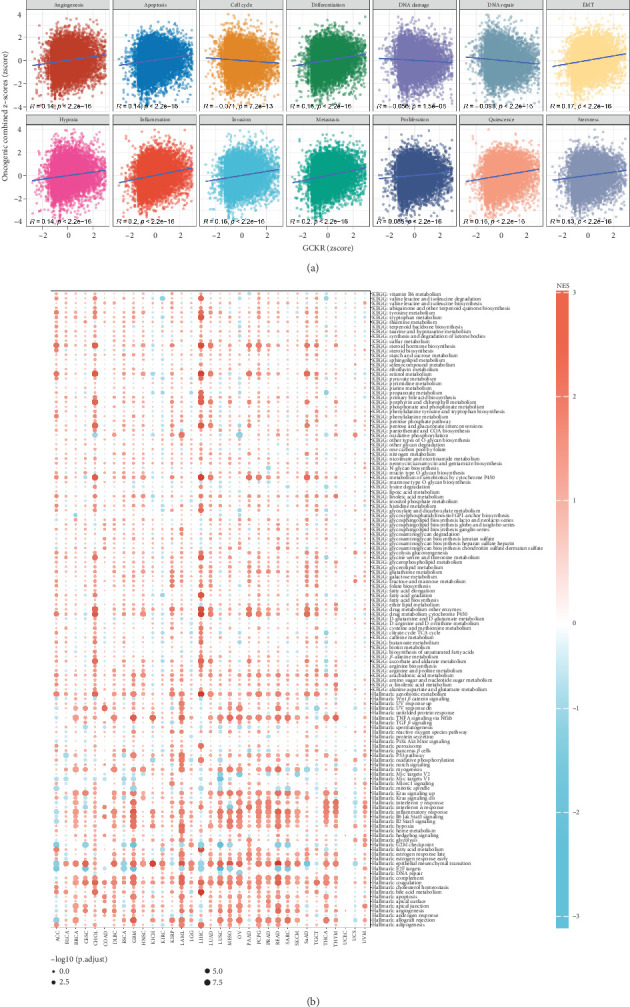
Pathway and mechanism analyses. (a) GCKR mRNA expression and 14 malignant phenotypes. (b) Enrichment differences in 50 HALLMARK and 83 metabolic gene sets.

We evaluated the relationship between GCKR and 14 cancer-related functional pathways by calculating combined *z*-scores for each gene set (e.g., angiogenesis, apoptosis, EMT, and DNA repair). Pearson's correlation analysis showed that GCKR was positively associated with most pathways but negatively correlated with cell cycle, DNA damage, and DNA repair (Figure 6a). Using the limma package, we performed differential gene expression analysis between the top and bottom 30% GCKR expression samples in each cancer type. GSEA revealed enrichment of cancer- and metabolism-related pathways in the GCKR high-expression group (Figure 6b). In CHOL, LIHC, and LAML, pathways such as xenobiotic metabolism, TNF-*α* signaling via NF-*κ*B, epithelial–mesenchymal transition, complement, and coagulation were significantly enriched in high GCKR expression groups.

### 3.7. Correlation Between GCKR and Functional Proteins

**Figure 7:**
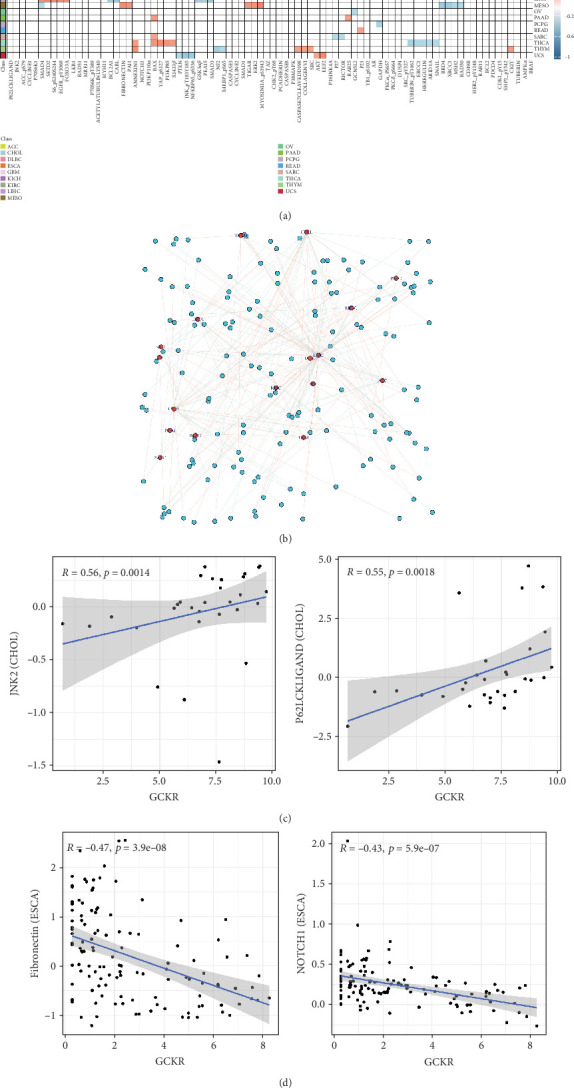
(a) Top 5 GCKR-correlated functional proteins (TCPA). Red: positive correlation; blue: negative; shade indicates correlation strength. (b) GCKR–protein network in cancer. Proteins significantly correlated with GCKR in (c) CHOL and (d) ESCA.

Using Spearman's correlation, we explored associations between GCKR and functional proteins involved in cancer progression.  Figure 7a shows the Top 5 positively and negatively correlated proteins across cancers. In CHOL, GCKR was positively correlated with JNK2 and P62LCKLIGAND and negatively correlated with MRE11 and RAD51.  Figure 7b displays the interaction network between GCKR and functional proteins across cancers, highlighting close associations in CHOL and LIHC. We further illustrated GCKR's top correlated and anticorrelated functional proteins in CHOL and ESCA (Figure 7c,d).

### 3.8. GCKR and Tumor Immunity

**Figure 8:**
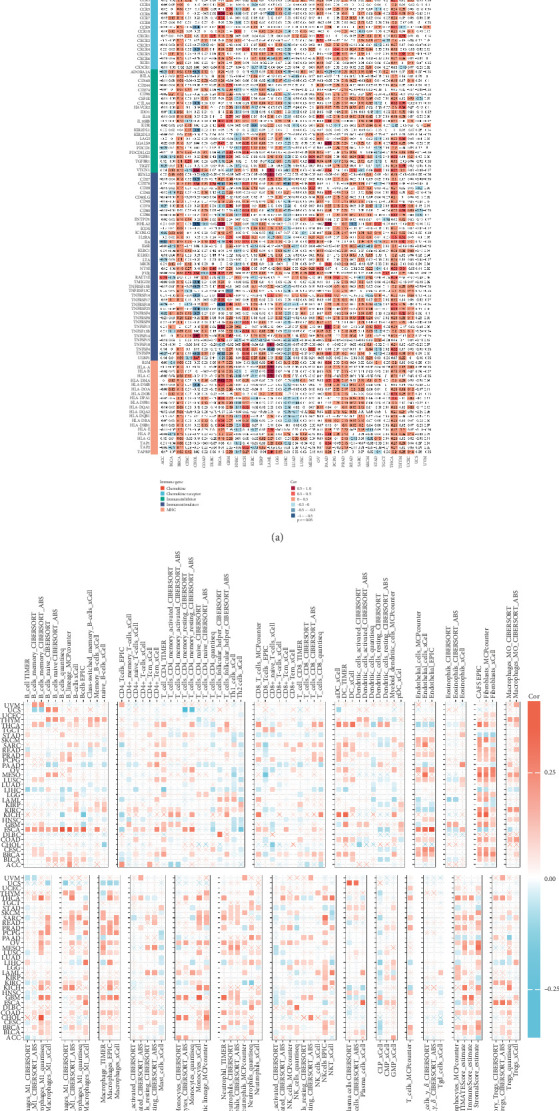
GCKR and cancer immunity. (a) GCKR and chemokines, receptors, immune suppressors/stimulators, and MHC genes. (b) GCKR and immune/stromal cell infiltration.

Given the crucial role of the immune microenvironment in tumor development, we analyzed the correlation between GCKR and immune-related genes and cells. GCKR was positively correlated with chemokines, chemokine receptors, immune stimulators, immune inhibitors, and MHC molecules in cancers such as BRCA, THCA, and PRAD. Conversely, negative correlations were observed in LIHC (Figure 8a). Using various immune infiltration algorithms, we found that GCKR expression was positively correlated with fibroblasts, macrophages, and monocytes in several cancers (Figure 8b), suggesting a role in modulating the tumor immune microenvironment.

### 3.9. Spatial Transcriptomic Analysis

**Figure 9:**
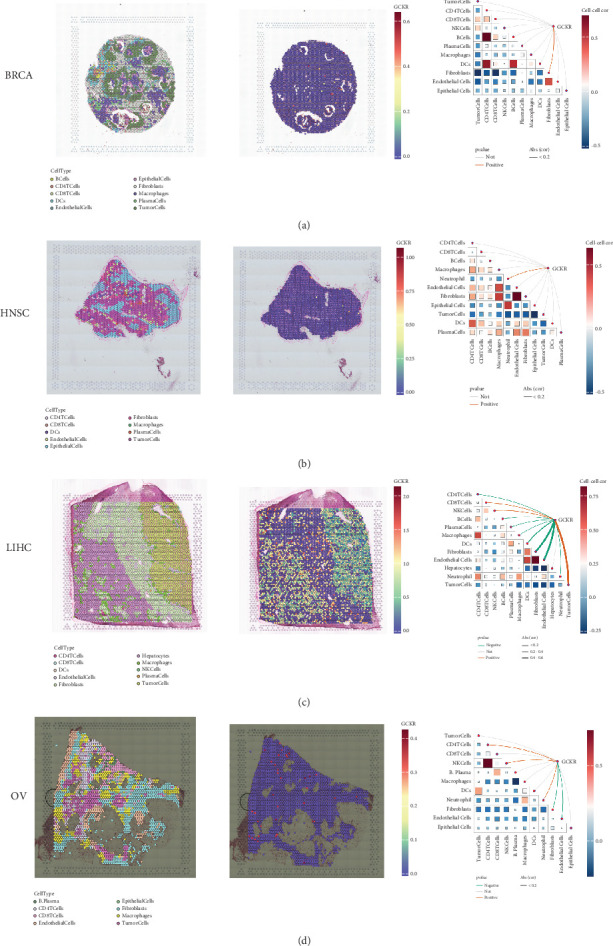
Analysis of GCKR-related chemotherapy resistance. (a, b) Correlation between drug sensitivity and GCKR mRNA expression in the GDSC1 and GDSC2 databases. (c) Compounds targeting GCKR. (d) Targeted compounds for GCKR in ACC, LUAD, PAAD, and PCPG. (e) Kaplan–Meier analysis: immunotherapy related to GCKR.

Using the SpatialFeaturePlot function in the Seurat package, we visualized the spatial enrichment of cell types. The color gradient indicated the relative abundance of specific cell types across regions. Quantitative analysis revealed significant correlations between regional cell abundance and GCKR expression (Figures 9a, 9b, 9c, and 9d). In BRCA, GCKR was positively associated with fibroblasts; in HNSC, with neutrophils; and in LIHC, with various immune cell types. In OV, GCKR was positively associated with both NK cells and neutrophils, suggesting a potential regulatory role in immune cell infiltration.

### 3.10. GCKR in Single-Cell Transcriptomic Data of Gastric Cancer

**Figure 10:**
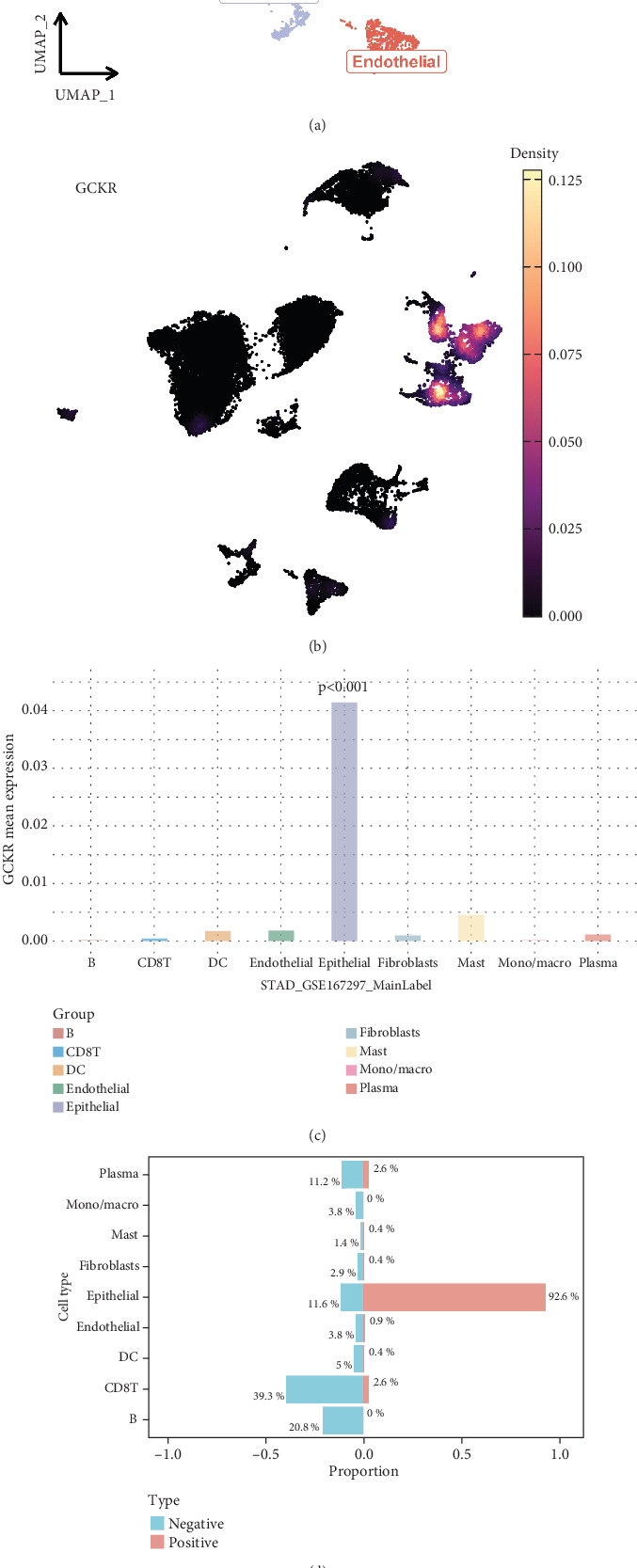
GCKR expression across four tumor types via spatial transcriptomics. (a–d) Cell-type maps show dominant cell characteristics and correlations between cell content and GCKR expression. (e) Pathway enrichment analysis of GCKR-positive cells shows upregulated metabolic and mitochondrial pathways, consistent across cell types.

Based on the GSE167297 dataset, UMAP visualization revealed clear clustering of heterogeneous cell subpopulations (Figure 10a). Single-cell gene expression analysis indicated distinct expression patterns of GCKR across cell types (Figure 10b). Quantitative comparisons showed significant heterogeneity in GCKR expression (Figure 10c). A binary classification model based on GCKR expression revealed differential cell type compositions, with mucus-secreting cells enriched in the GCKR-positive group (Figure 10d). Pathway enrichment analysis demonstrated that GCKR-positive cells exhibited upregulated metabolic and mitochondrial pathways, which were consistent across cell types (Figure 10e). These findings suggest that GCKR may enhance metabolic reprogramming, contributing to tumor adaptation in the gastric cancer microenvironment.

### 3.11. Correlation Analysis Between GCKR and CAF Infiltration

**Figure 11:**
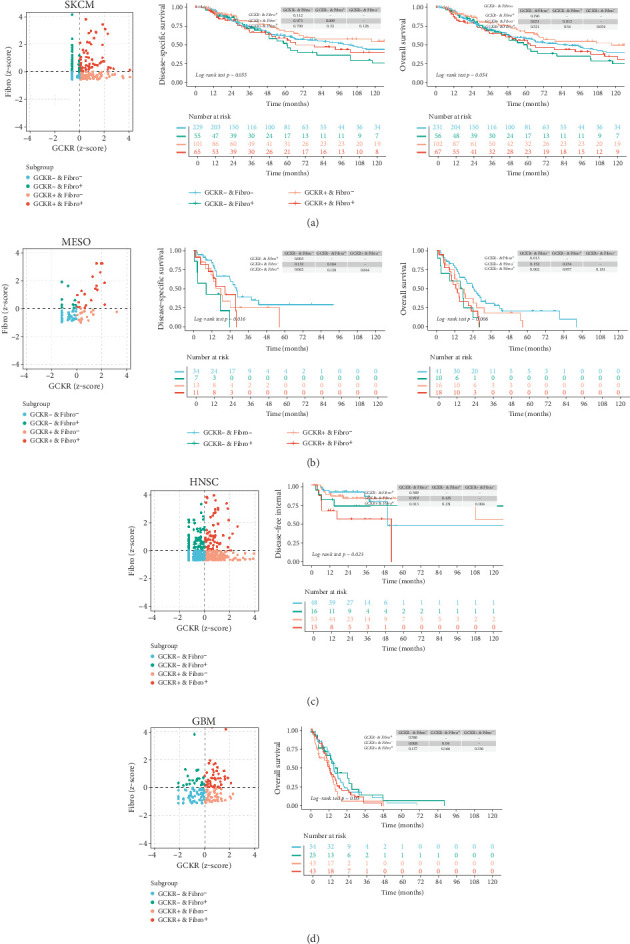
Correlation analysis between GCKR and CAF infiltration. The *z*-score scatter plots, Kaplan–Meier survival curves, and log-rank test results for (a) SKCM, (b) MESO, (c) HNSC, and (d) GBM, respectively.

We combined immune infiltration data from TIMER2.0 with transcriptomic data to examine the relationship between GCKR expression in particular cell populations and patient survival or clinical outcomes. Previous analyses have indicated a possible link between GCKR and CAFs. Therefore, we investigated the relationship among GCKR expression, the degree of CAF infiltration, and patient prognosis. Our results suggest that, in SKCM (Figure 11a), MESO (Figure 11b), HNSC (Figure 11c), and GBM (Figure 11d), patients with both low CAF infiltration and low GCKR expression tend to have a better prognosis. This may imply a potential role for GCKR in CAF function or in CAF-mediated remodeling of the tumor microenvironment.

### 3.12. GCKR and Drug Sensitivity and Immunotherapy

**Figure 12:**
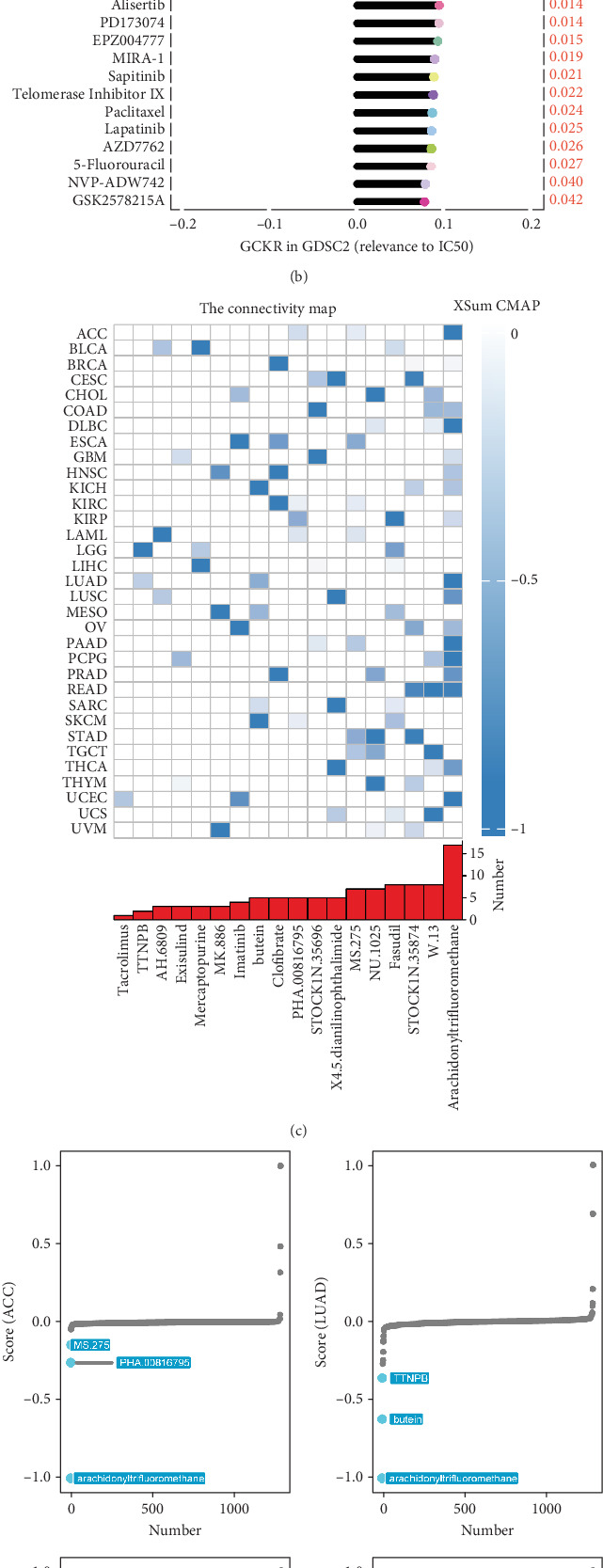
GCKR in gastric cancer single-cell data. (a) Dimensional reduction of GSE167297. (b) Single-cell GCKR expression. (c) Expression across cell types. (d) Cell type proportions in positive versus negative groups. (e) Scoring differences between positive and negative groups.

Using the GDSC database, we analyzed the impact of GCKR on commonly used chemotherapeutic drugs in cancer.  Figure 12a,b shows that GCKR expression is positively correlated with the IC50 of most drugs and only negatively correlated with refametinib and PD0325901. This indicates that high GCKR expression may reduce tumor sensitivity to most chemotherapeutic agents while increasing sensitivity to refametinib and PD0325901. Therefore, tumors with high GCKR expression may respond better to these two drugs. Next, we used the CMap database to identify drugs that affect GCKR activity in cancer. We found that in pan-cancer analysis, compounds such as arachidonyltrifluoromethane, W.13, and STOCK1N.35874 significantly inhibit GCKR activity (Figure 12c). Specifically, arachidonyltrifluoromethane significantly suppresses GCKR activity in ACC, LUAD, and PAAD, while NU.1025 and STOCK1N.35874 show a strong inhibitory effect in STAD (Figure 12d). We then conducted a clinical cohort analysis to assess the potential of GCKR as a predictor of response to immunotherapy. Moreover, survival analysis revealed that patients with high GCKR expression had significantly longer survival times (Figure 12e).

## 4. Discussion

In previous studies, we have revealed the important role of metabolism and immune-related molecules in gastric cancer [25, 27]. Building on this, we further explored the influence of GCKR in this process. This study presents the first comprehensive pan-cancer analysis of the expression patterns, mutational characteristics, and mechanistic implications of GCKR across multiple cancer types, with a particular emphasis on how metabolic regulation intersects with immune remodeling in gastric cancer. GCKR, a key regulator of glucose metabolism, is found to be downregulated in most tumor tissues, with particularly marked differences in BRCA and CHOL. Conversely, GCKR tends to be upregulated in certain tumors such as KIRP and LUAD. This variation suggests that GCKR may play a dual regulatory role depending on the tumor context, potentially contributing to the modulation of complex metabolic–immune networks.

The tumor immune microenvironment is widely recognized as a critical factor in tumor development [28]. Metabolic reprogramming is known to facilitate its formation [29], and glucose metabolism constitutes a significant component of cancer metabolism [30]. Numerous prior studies have highlighted the importance of glucose metabolism in malignant tumors [31]. GCKR plays a fundamental role in human glucose metabolism and is involved in various pathophysiological processes [10], yet its role in cancer has seldom been explored. Our findings suggest that loss of GCKR disrupts glucose homeostasis, thereby intensifying glycolytic flux and nutrient competition, which may contribute to the establishment of an immune-cold microenvironment.

We analyzed the expression and mutation profile of GCKR in cancer, along with its associations with clinical features, immune infiltration, and drug sensitivity. Spatial transcriptomic data were used to assess the relationship between GCKR and immune cells. We then focused specifically on the expression patterns of GCKR in gastric cancer and validated its expression experimentally. Importantly, single-cell and spatial transcriptomic analyses revealed that GCKR-positive subpopulations are enriched for mitochondrial and metabolic pathways, and their localization within tumor niches provides a mechanistic link between metabolic heterogeneity and immune exclusion.

Our findings indicate that GCKR expression is lower in most tumors compared with normal tissues, notably in BRCA and CHOL, but elevated in cancers such as KIRP and LUAD. This differential pattern suggests GCKR may serve as a potential biomarker for cancer diagnosis. Furthermore, the high mutation rates of GCKR observed in SARC and UCEC suggest a possible role in their tumorigenesis. Of particular note, missense mutations were the most common type of SNV identified in GCKR, hinting at possible functional alterations in the GCKR protein that may contribute to tumor progression. These findings imply that both expression and mutational alterations of GCKR could reprogram tumor metabolism and immune signaling in a context-dependent manner, warranting further mechanistic exploration.

Cancer staging and diagnosis are critical for guiding clinical decisions [32]. Our analysis reveals that GCKR expression correlates with tumor pathological grade. As malignancy increases, GCKR expression rises in cancers such as KIPAN, KIRC, and PAAD. Expression is also associated with TNM staging in several cancers, including KIPAN, ESCA, and PAAD. Given the strong link between tumor grade, staging, and prognosis [33], GCKR may serve as a useful marker for early diagnosis. Additionally, GCKR expression in THCA was found to be age-related, which may reflect age-associated metabolic regulation [34]. The observed associations between GCKR and tumor stage or grade suggest that it may help predict cancer aggressiveness and metastatic risk [35], offering potential value in clinical decision-making. However, these descriptive associations should be interpreted with caution, and their mechanistic underpinnings—particularly the link to metabolic rewiring and immune evasion—require further investigation.

Early diagnosis is equally vital. We found that GCKR exhibits relatively high AUC values in many cancers. In CHOL and LIHC, the AUC values exceed 0.85, suggesting GCKR may serve as a diagnostic marker for CHOL and LIHC. Our findings also suggest that GCKR is a prognostic risk factor in cancers such as GBMLGG and GBM. In further cohort analyses, GCKR was a risk factor for BRCA and BLCA, while appearing protective in STAD and LIHC. These findings raise the possibility that GCKR may serve as a prognostic biomarker in specific cancers.

GCKR was found to correlate positively with several gene sets involved in tumor functional states. In CHOL, GCKR was associated with various signaling pathways, indicating a potentially significant role in tumor progression. Moreover, pathways such as xenobiotic metabolism, TNF-*α* signaling via NF-*κ*B, epithelial–mesenchymal transition, complement activation, and coagulation were enriched in GCKR-high groups. This implies that GCKR may influence tumor progression by regulating these pathways. Notably, GCKR expression also correlated strongly with functional cancer-related proteins in several cancers. For example, in CHOL, GCKR showed strong positive correlations with JNK2 and P62LCKLIGAND, suggesting that GCKR might influence tumor survival and apoptosis by modulating these pathways—findings consistent with prior reports [36, 37]. These observations open new avenues for understanding GCKR's function in cancer.

Analysis of the tumor immune microenvironment showed significant correlations between GCKR and chemokines, their receptors, immune suppressive and stimulatory factors, and MHC molecules across several cancers. This suggests that GCKR may influence immune cell recruitment, activation, and function, thereby playing a role in tumor development. Interestingly, the nature of GCKR's associations with immune factors varied between cancers, indicating possible tumor-specific immunomodulatory roles. GCKR mRNA expression was positively associated with fibroblasts, macrophages, and monocytes in many cancers, pointing to a potential role in shaping the immune microenvironment and contributing to cancer progression [38].

We further explored how GCKR affects drug sensitivity. Data from the GDSC database showed that higher GCKR expression reduces sensitivity to several drugs. This implies that suppressing GCKR may improve drug responsiveness, suggesting its potential as a therapeutic target. Compounds such as arachidonyltrifluoromethane, W.13, and STOCK1N.35874 were identified as significant GCKR inhibitors, indicating their promise for targeted therapy development. Of particular interest, high-GCKR tumors showed selective sensitivity to MEK inhibitors, including refametinib and PD0325901. Docking and molecular dynamics simulations confirmed favorable binding profiles of these agents with GCKR, underscoring their translational potential. While pharmacological studies are beyond the scope of this work, our findings nominate GCKR as a biomarker that may guide MEK inhibitor–based therapies in future clinical applications.

Spatial transcriptomic analysis confirmed GCKR's associations with immune cells. The nature of these associations varied across cancers, highlighting the immune-specific role of GCKR. Its interaction with immune cells suggests a potential role in modulating the tumor immune microenvironment, possibly affecting tumor initiation and progression through regulation of immune cell function. Single-cell transcriptomic analysis of gastric cancer revealed marked heterogeneity in GCKR expression across cell types. Mucinous cell subsets were enriched in GCKR-positive groups, which also showed heightened metabolic regulation and mitochondrial activity. These findings imply that GCKR may enhance metabolic reprogramming capacity and promote gastric cancer progression.

Taken together, our results suggest that GCKR is not only a central regulator in glucose metabolism but may also contribute to cancer development through metabolic–immune interactions. By shaping tumor metabolism and immune infiltration, GCKR emerges as both a biomarker and a potential therapeutic guide. Its tissue-specific expression and multifaceted roles provide a theoretical foundation for developing precision treatment strategies, including biomarker-driven immunotherapy and MEK inhibitor–based interventions. Future studies should validate these mechanisms experimentally and prospectively test GCKR-guided therapeutic approaches.

## 5. Limitations

Despite employing multiomics integration to systematically explore GCKR's roles across cancers, several limitations should be acknowledged. First, the analyses relied primarily on retrospective datasets such as TCGA and GTEx, which may introduce inherent heterogeneity and potential selection bias. Second, although GCKR expression was experimentally validated in gastric cancer, comparable validation in other tumor types was not performed and will be necessary to strengthen the generalizability of our conclusions. Third, while our findings provide mechanistic hypotheses linking GCKR to metabolic reprogramming and immune regulation, the precise molecular pathways remain incompletely defined. Future work, including gene knockout models, metabolic flux tracing, and in vivo validation, will be critical to delineate the causal mechanisms. Finally, although our drug-sensitivity analysis highlighted refametinib and PD0325901 as potential therapeutic candidates, more extensive pharmacological and preclinical studies are required before clinical translation can be considered.

## 6. Conclusion

This multiomics and experimentally validated study identifies GCKR as a key link between metabolic reprogramming and immune regulation in gastric cancer. Low GCKR expression aligns with an immune-cold microenvironment—marked by reduced cytotoxic T cell infiltration and suppression of antigen presentation/interferon signaling—and associates with poorer outcomes, while spatial and single-cell analyses delineate its compartment-specific heterogeneity. Clinically, GCKR shows promise as an integrative biomarker for diagnosis, prognosis, and treatment stratification; notably, drug-response profiling suggests that tumors with higher GCKR expression may be selectively vulnerable to MEK inhibition, nominating refametinib and PD0325901 as testable therapeutic options. These findings provide a rationale for GCKR-guided patient selection and prospective evaluation of MEK inhibitor–based strategies in gastric cancer.

## Data Availability

The data that support the findings of this study are available from the corresponding authors upon reasonable request.

## Supporting Information

Supporting Information 1 Figure S1: Expression landscape of GCKR across cancers and human organs. (A) GCKR expression in tumor versus normal tissues (TCGA). (B) Distribution of GCKR across organs. Note: ⁣^∗∗∗∗^*p* < 0.0001, ⁣^∗∗∗^*p* < 0.001, ⁣^∗∗^*p* < 0.01, and ⁣^∗^*p* < 0.05.

Supporting Information 2 Figure S2: Additional machine learning validation of GCKR in gastric cancer. (A) Boruta feature importance. (B) Lasso cross-validation error curve. (C) Random forest error rate curve. (D) SVM-RFE feature ranking. (E) XGBoost feature importance.

Supporting Information 3 Figure S3: Associations of GCKR expression with clinicopathological parameters. (A) GCKR and pathological grade. (B) GCKR and patient sex. Note: ⁣^∗∗∗∗^*p* < 0.0001, ⁣^∗∗∗^*p* < 0.001, ⁣^∗∗^*p* < 0.01, and ⁣^∗^*p* < 0.05.
