# Supplementary figures and images for: Glucokinase Regulatory Protein (GCKR) Links Metabolic Reprogramming With Immune Exclusion: Insights From a Pan-Cancer Analysis and Gastric Cancer Validation
Source: Hum Mutat. 2025 Nov 5;2025:4240223. doi: 10.1155/humu/4240223 (PMC12611472; doi:10.1155/humu/4240223)

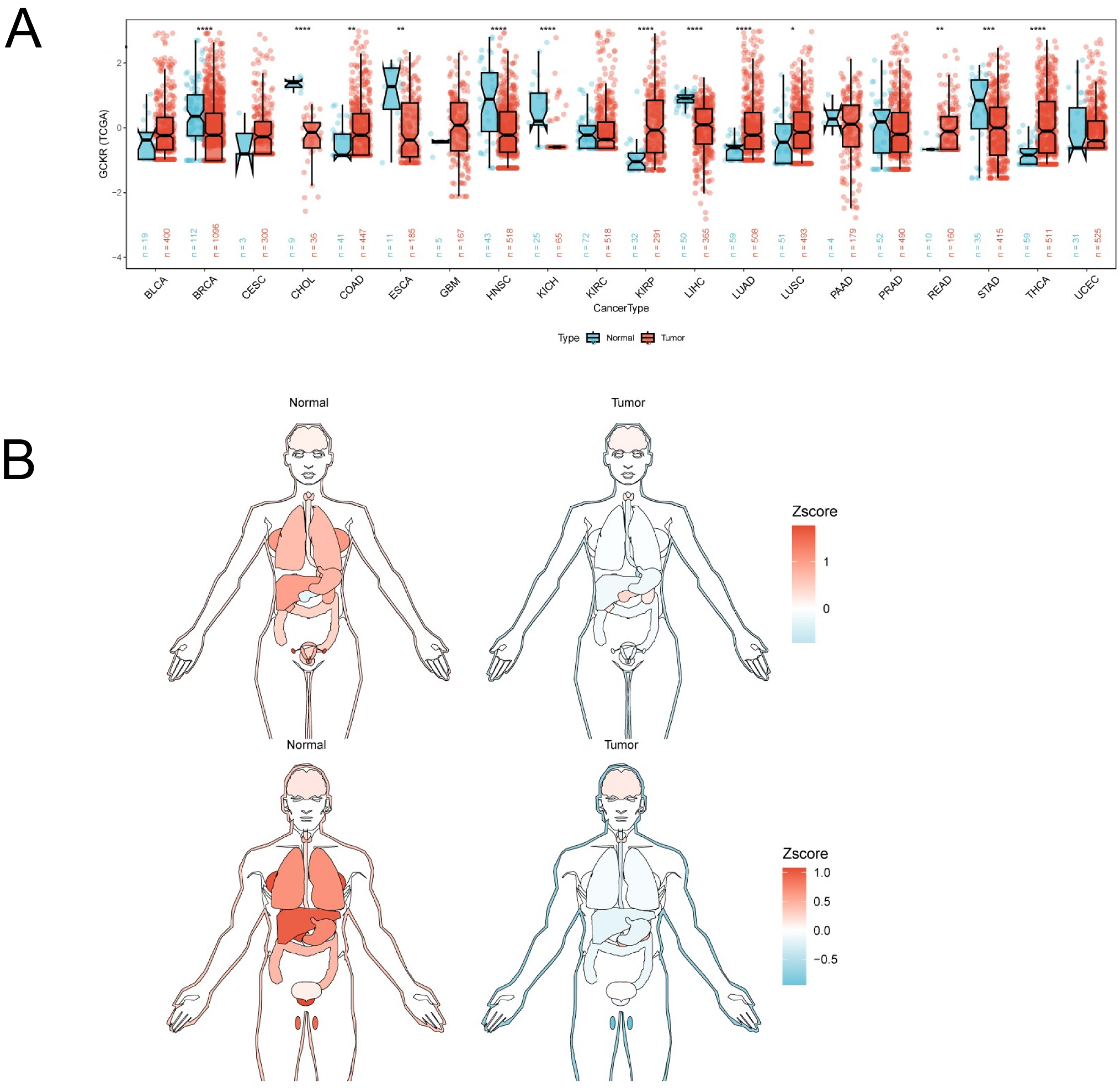

Supplement: Supporting Information 1 — Figure S1: Expression landscape of GCKR across cancers and human organs. (A) GCKR expression in tumor versus normal tissues (TCGA). (B) Distribution of GCKR across organs. Note: ⁣∗∗∗∗p < 0.0001, ⁣∗∗∗p < 0.001, ⁣∗∗p < 0.01, and ⁣∗p < 0.05. [file 4240223.f1.tif]

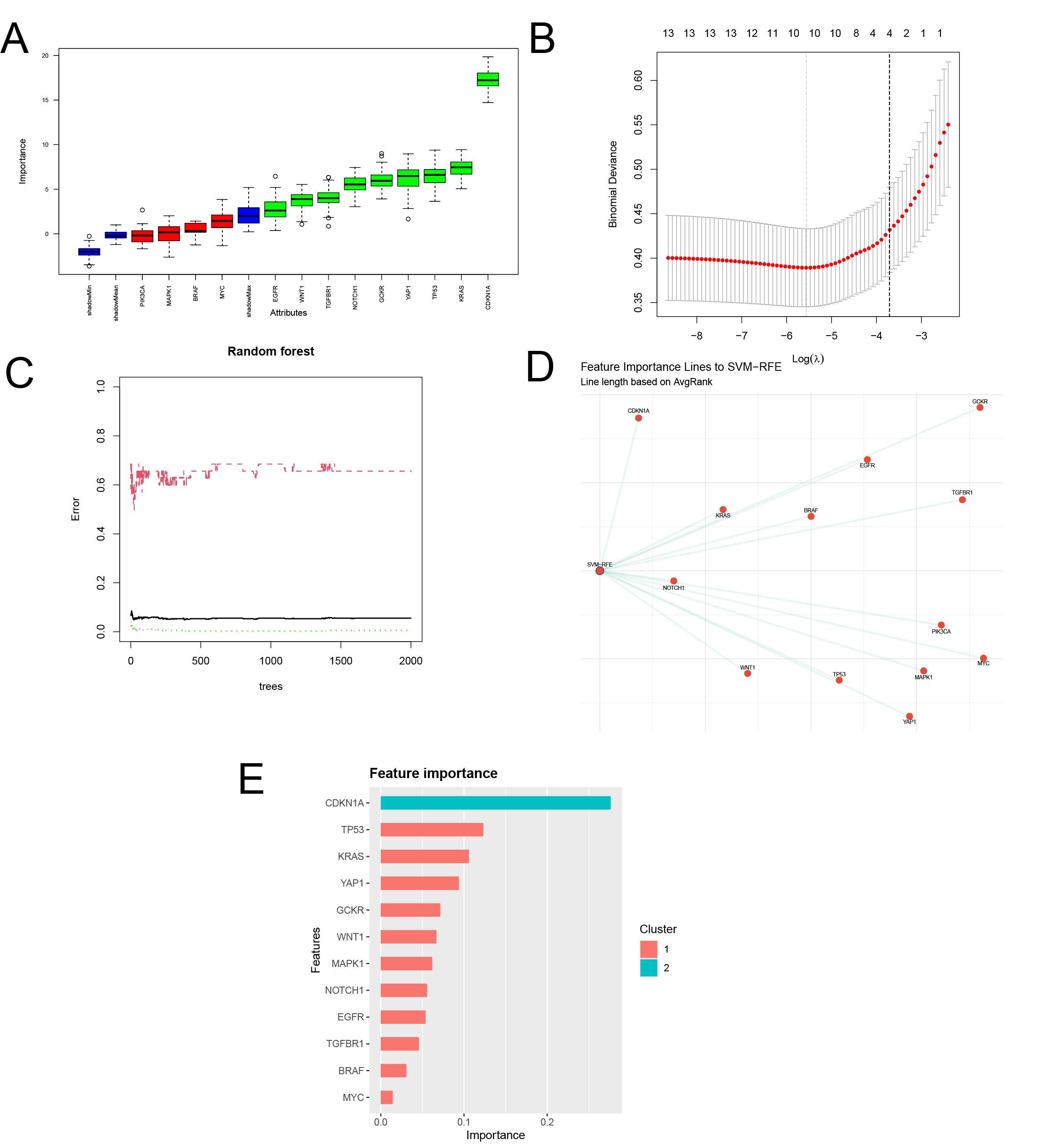

Supplement: Supporting Information 2 — Figure S2: Additional machine learning validation of GCKR in gastric cancer. (A) Boruta feature importance. (B) Lasso cross-validation error curve. (C) Random forest error rate curve. (D) SVM-RFE feature ranking. (E) XGBoost feature importance. [file 4240223.f2.tif]

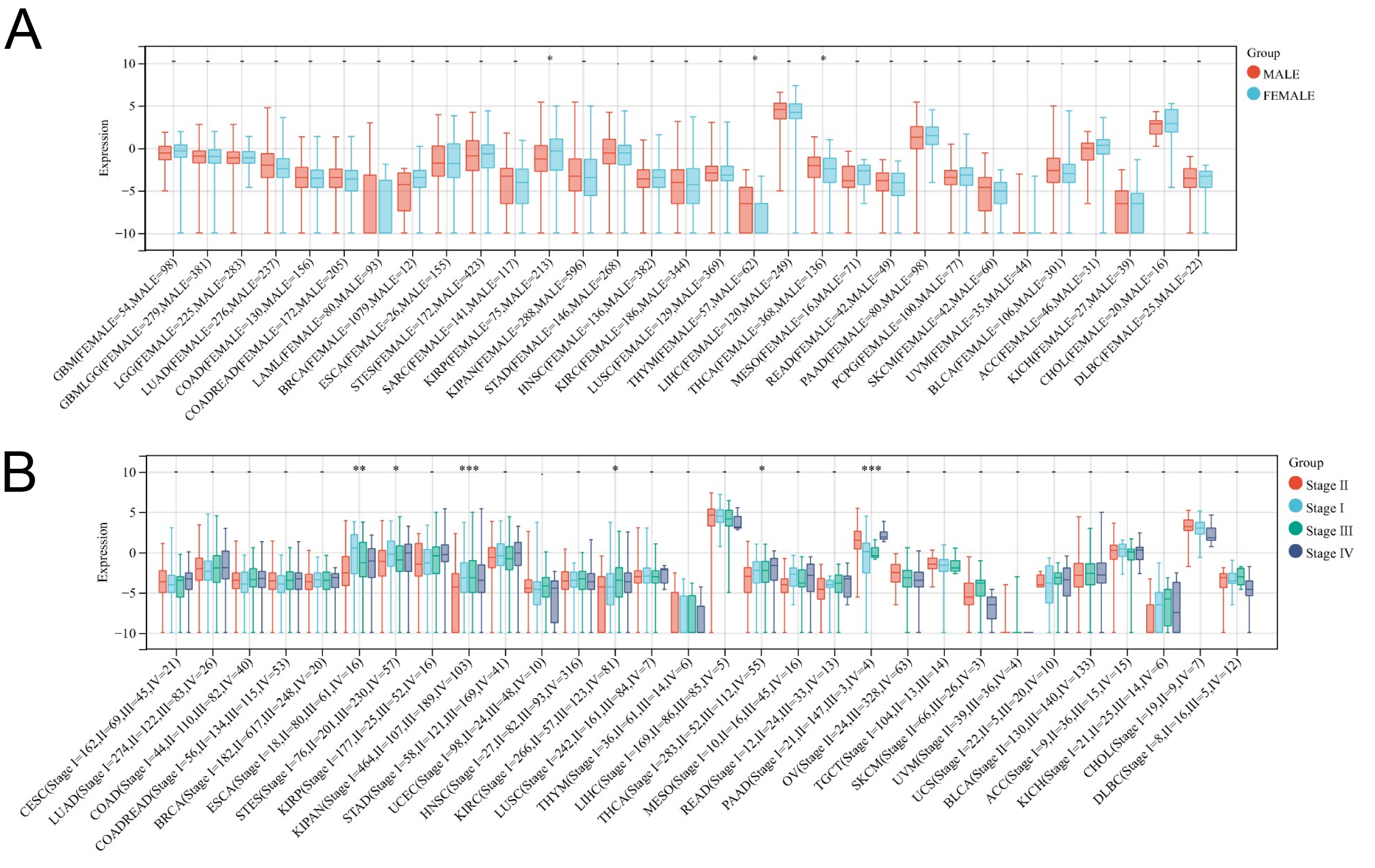

Supplement: Supporting Information 3 — Figure S3: Associations of GCKR expression with clinicopathological parameters. (A) GCKR and pathological grade. (B) GCKR and patient sex. Note: ⁣∗∗∗∗p < 0.0001, ⁣∗∗∗p < 0.001, ⁣∗∗p < 0.01, and ⁣∗p < 0.05. [file 4240223.f3.tif]
